# Book Notices

**Published:** 1870-05

**Authors:** 


### Book Notices.

Modern Therapeutics, a Compendium of Recent Formulæ and Specific Therapeutical Directions. By GEO. H. NAPHEYS, A.M., M.D.; one of the Editors of the *Half-Yearly Compendium of Medical Science*, etc., etc. Philadelphia: S. W. Butler, M.D. 1870.

This is a duodecimo volume of 390 pages, published in fair style, and filled with an abundance of formulæ or prescriptions for the various ills that flesh is heir to. This book will prove a great convenience to all such members of the profession as depend on formulas, instead of a knowledge of the principles that govern the action of medicines, for guidance in the treatment of disease.

---

The Preventive Obstacle, or Conjugal Onanism, the Dangers and Inconveniences to the Individual, to the Family, and to Society, of Frauds in the Accomplishment of the Generative

Functions. By L. F. E. BERGERET, Physician-in-Chief to the Arbois Hospital, Genoa. Translated from the Third French Edition, by P. DEMARMON, M.D. New York: Turner & Mignard. 1870.

This is a neatly published 12mo. of 182 pages. It is largely filled with the relation of cases, not half of which appear to have any bearing on the subjects they are adduced to illustrate. It is written in a style altogether too exaggerated and sensational to merit the name of a scientific monograph.

---

A Practical Treatise on the Diagnosis, Pathology, and Treatment of Diseases of the Heart. By AUSTIN FLINT, M.D.; Professor of the Principles and Practice of Medicine, and of Clinical Medicine, in the Bellevue Hospital Medical College, etc. Second Edition; thoroughly Revised and Enlarged. Philadelphia: Henry C. Lea. 1870. For sale by S. C. Griggs & Co., Chicago. Pp. 550. Price $4.

The high estimation in which all the works of Dr. Flint are held by the profession, and the well-known merits of the first edition of this treatise, render any commendation of this, second edition, unnecessary. It is worthy of a place in every medical library.

---

The Indigestions, or Diseases of the Digestive Organs, Functionally Treated. By THOMAS KING CHAMBERS, Honorary Physician to H. R. H. the Prince of Wales; Senior Consulting Physician and Lecturer on the Practice of Medicine, at St. Mary's Hospital, etc., etc. Third American Edition, Revised. Philadelphia: Henry C. Lea. 1870. Pp. 383. Price $3.

This is a new edition of Chambers' well-known work on functional disorders of the stomach. It contains many additional cases, with such modifications in the original text as was suggested or required for their insertion. The edition is exclusively American, a third edition not yet having been called for in England. It is published in excellent style.

